# Toward health system strengthening in low- and middle-income countries: insights from mathematical modeling of drug supply chains

**DOI:** 10.1186/s12913-020-05549-z

**Published:** 2020-08-24

**Authors:** Abdulrahman Jbaily, Isabelle Feldhaus, Benjamin Bigelow, Leila Kamareddine, Mieraf Taddesse Tolla, Marion Bouvier, Mizan Kiros, Stéphane Verguet

**Affiliations:** 1 Department of Global Health and Population, Harvard T.H. Chan School of Public Health, 655 Huntington Ave, Boston, 02115 MA USA; 2 Hospital IQ, 55 Chapel St Suite 102, Newton, 02458 MA USA; 3 Université Lille 2 Droit Et Santé, Lille, France; 4 Ethiopian Health Insurance Agency, 1 bis Rue Georges Mandel, Addis Ababa, 59000 Ethiopia

**Keywords:** Drug supply chain, Mathematical modeling, Health system modeling, Value for money, Health system strengthening, Low- and middle-income countries

## Abstract

**Background:**

Global health priority setting increasingly focuses on understanding the functioning of health systems and on how they can be strengthened. Beyond vertical programs, health systems research should examine system-wide delivery platforms (e.g. health facilities) and operational elements (e.g. supply chains) as primary units of study and evaluation.

**Methods:**

We use dynamical system methods to develop a simple analytical model for the supply chain of a low-income country’s health system. In doing so, we emphasize the dynamic links that integrate the supply chain within other elements of the health system; and we examine how the evolution over time of such connections would affect drug delivery, following the implementation of selected interventions (e.g. enhancing road networks, expanding workforce). We also test feedback loops and forecasts to study the potential impact of setting up a digital system for tracking drug delivery to prevent drug stockout and expiration.

**Results:**

Numerical simulations that capture a range of supply chain scenarios demonstrate the impact of different health system strengthening interventions on drug stock levels within health facilities. Our mathematical modeling also points to how implementing a digital drug tracking system could help anticipate and prevent drug stockout and expiration.

**Conclusion:**

Our mathematical model of drug supply chain delivery represents an important component toward the development of comprehensive quantitative frameworks that aim at describing health systems as complex dynamical systems. Such models can help predict how investments in system-wide interventions, like strengthening drug supply chains in low-income settings, may improve population health outcomes.

## Background

Essential medicines are drugs identified to satisfy the priority health needs of populations. They are selected based on the burden of disease, public health relevance, drug safety and efficacy, and the comparative cost and cost-effectiveness across drugs [1]. National lists of essential medicines at the country level are intended to identify those medicines to be available for delivery within health services and facilities at all times in adequate amounts, in the appropriate dosage forms, with assured quality, and at an affordable price [1]. However, essential medicines remain unavailable and inaccessible to many around the world, particularly in low- and middle-income countries (LMICs) [2–6]. Stockouts of essential medicines at the hospital and clinic levels are a widely acknowledged issue in resource-constrained settings that limit access and have significant negative impacts on morbidity, mortality, and disease burden [7–9]. Preventing drug stockouts and promoting greater access to essential medicines highly depends on well-functioning supply chains that can efficiently distribute drugs from manufacturers to health facilities to patients at the point of service delivery.

Public sector supply chains for essential medicines face a number of important challenges in LMICs. Most governments in these settings operate under distribution models in which a central actor procures drugs and distributes them to health clinics using a government-owned transportation system [10]. Root causes of stockouts at health clinics under these structures include diffuse accountability driving fragmentation of responsibility and governance, uncertainties in financing, unnecessary levels of complexity that negatively affect communication and decision-making, long resupply intervals, low commitment to funding operating costs, lack of supply chain planning data, weak planning capacity, and lacking incentives to improve supply chain performance [10]. Therefore, modeling of pharmaceutical supply chains has been used as a tool to understand and address challenges of specific health systems, including for vaccine delivery [11–14]. These supply chains can be managed in a variety of ways, such as ‘push’ systems - where the product is ‘pushed’ towards end users based on projected demand - and ‘pull’ systems - where real-time demand drives production levels [15]. Many other supply chain methodologies exist, such as ‘just-in-time’ (JIT) inventory management, which aims to increase efficiency by minimizing the inventory on hand [16]. Current approaches have not yet achieved sufficient integration of health system factors along the pharmaceutical supply chain to inform improvements and interventions [17].

Studying health system strengthening (HSS) interventions rather than single technical interventions is needed to help policymakers choose what may be good value for money investments for health systems [18]. HSS interventions importantly include pharmaceutical procurement and supply chain management. Mathematical models including simple dynamical systems and simple systems of differential equations can be useful in describing the dynamics of supply chain systems as a function of specified parameters, which can provide key insights for informed decision-making. These parameters can account for intrinsic features of the health system and allow for end-to-end integration of the supply chain, connecting manufacturing technologies, government procurement, transport to health facilities, and health provider behavior to patient receipt and health. Here, we present a general model of a supply chain that is governed by specific parameters pertaining to the health system in general, such as the condition and quality of roads, the size of the health workforce, the levels of immunization coverage, etc. The developed model can then be used as part of a greater modeling framework that describes the whole health system. Here, we investigate through our parametric modeling approach how different health system strengthening policies, not always specific to supply chain delivery, can impact on drug levels and we emphasize the case of one supply chain specific intervention pertaining to the installation of a digital system for tracking medicine stocks.

## Methods

We develop a mathematical model where essential medicines go through multiple stages before being available to the public and patients in need. The multistage process is complex and can vary from one country to another, but in general it starts with manufacturers who send the drugs to the procurers who in turn send them to storage warehouses. Drugs are then distributed to the health facilities, where patients and individuals in need can collect them. There also can exist intermediate levels along this multistage process in provinces or districts, with private wholesalers and non-governmental organizations (NGOs) for example.

**Fig. 1:**
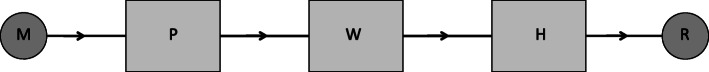
Simplified schematic depicting a supply chain of essential medicines in a low- and middle-income country setting. M: manufacturers, P: procurers, W: warehouses, H: health facilities, R: recipients of drugs (i.e. patients)

The variability in stock size available at health facilities can be sensitive to many factors because of the multistage process involved in drug delivery which we described above. Such factors are not necessarily specific to the supply chain system and can belong to the broader health system more generally. For example, immunization programs adopted by a country can affect patient demand for drugs, road availability and conditions can influence drug transportation times, and the health workforce can be impacting on the capacity and frequency of shipments. Moreover, there can be disease-specific factors, such as the influence of seasonality, when, for example, during specific seasons, some diseases (e.g. malaria, rotavirus) may be more prevalent than others. Accounting for all these effects requires a complex approach that heavily depends on country-specific datasets. Here, we follow a simple and interpretable approach that is easily generalizable to multiple LMICs. We develop a mathematical model that utilizes health system characteristics to describe the interactions among manufacturers, procurers, warehouses, health facilities and drug recipients (Fig. 1). We use our analytical model to emphasize the importance of the connections between the different components of the whole health system; in particular, here we intend to monitor stock levels as affected by a range of government policies. Furthermore, we use the model to investigate the effect of the installation of an automated system to track medicines, and show how such an intervention specific to pharmaceutical procurement and supply chain management could potentially improve drug delivery.

### Mathematical model

We present a simple mathematical model composed of ordinary differential equations intended to capture the variations in stocks and flows involved in the drug supply chain. The model is composed of three compartments, where drug stock levels are computed: procurers (P), warehouses (W), and health facilities (H). The manufacturers ‘M’ and drug recipients ‘R’ serve as an input to the procurers and as an output from the health facilities compartments, respectively (Fig. 1). The evolution of the stock levels for the compartments of interest can be determined by the following set of differential equations:

1 $$  \frac{d Y_{P}(t)}{dt}=F_{MP}\left(t-\tau_{MP}\right)-F_{PW}(t),  $$

2 $$  \frac{d Y_{W}(t)}{dt}=F_{PW}\left(t-\tau_{PW}\right)-F_{WH}(t),  $$

3 $$  \frac{d Y_{H}(t)}{dt}=F_{WH}\left(t-\tau_{WH}\right)-D(t),  $$

where *Y*_*P*_(*t*), *Y*_*W*_(*t*), and *Y*_*H*_(*t*) are the drug stock levels for the procurers, warehouses and health facilities, respectively. *F*_*MP*_(*t*) is the flow rate of drugs (drug quantity per time) from manufacturers to procurers. Similarly, *F*_*PW*_(*t*) and *F*_*WH*_(*t*) are drug flow rates from procurers to warehouses, and from warehouses to health facilities, respectively. For simplicity, we refer to such quantities as shipment functions. *D*(*t*) is a demand function that describes acquisition of drugs by the recipients/patients from health facilities; it can also be expressed as a rate. Evidently, modeling of the demand function *D*(*t*) is not fixed and is case-dependent. *τ*_*MP*_ is the time it takes a shipment from the manufacturers to reach the procurers; we define it as shipment transportation time. Likewise, *τ*_*PW*_ and *τ*_*WH*_ are shipment transportation times from procurers to warehouses and from warehouses to health facilities, respectively. Transportation times *τ*_*MP*_, *τ*_*PW*_, and *τ*_*WH*_ can characterize the different delays in the supply chain system.

**Fig. 2:**
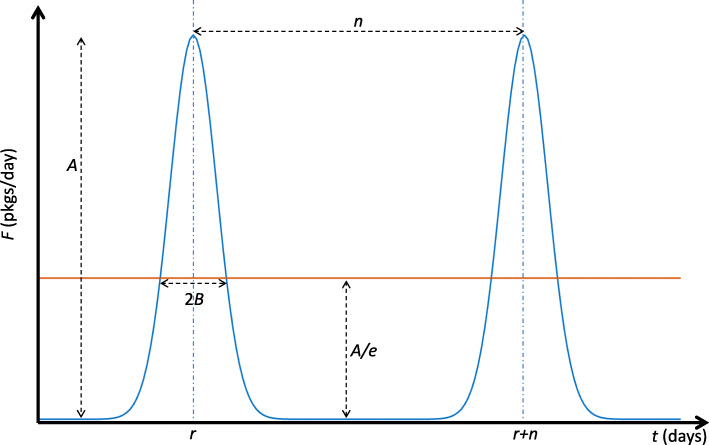
Mathematical features that describe the shipment function. *B* is defined at the value of *F* equal to *A*/*e*

Although shipments occur discretely, we model the shipment functions (*F*_*MP*_,*F*_*PW*_,*F*_*WH*_) with a Gaussian function. Such choice offers a smooth mathematical function that allows for the control of the size and duration for loading and unloading of shipments across the different compartments of the supply chain model (Fig. 2):

4 $$  F(t)=A e^{-\frac{(t-r)^{2}}{B^{2}}},  $$

where *A* is the amplitude of the function, *B* is related to the duration over which the shipment occurs and *r* is the time at which *F* reaches its peak; if shipments were to be modeled as discrete jumps, we take the limit *B*→0. Such shipment (e.g. drug packages) can be repeated with a certain periodicity or frequency, every *n* days for example (Fig. 2). The shipment size (*SS*) (e.g. number of drug packages or drug tonnage) is equal to the area under the curve, and can be expressed as:

5 $$  SS \approx AB\sqrt{\pi}~(pkgs).  $$

A summary of all the model parameters defined in this paper is given in Additional file 1.

### Effect of government interventions

The simple supply chain model which we exposed previously offers a pragmatic way for governments to asses how certain interventions can affect the performance of their essential drug supply chain. To improve performance, governments can implement a variety of policies. Patient drug demand *D*(*t*) for example can be decreased in the long term by investing in prevention programs (e.g. immunization programs, behavioral risk factor control) to lower the population needs for treatment interventions (e.g. seeking care for drugs). Transportation times can be reduced by improving the quality, design, and layout of the roads, or by increasing the number of warehouses and health facilities across the country, or by improving their distributions. Shipments can be scheduled more frequently among procurers, warehouses and health facilities by recruiting more personnel to conduct the delivery. In addition, the government can invest in purchasing additional transportation trucks to move higher loads of drugs and consequently increase shipment sizes or frequency. Certain policies can lead to an improvement of more than one factor. For example, building train rails can lead to shorter transportation times and larger shipments. However, depending on the country’s abilities, executing one policy or intervention might be easier or more cost-effective than executing another. Table 1 summarizes how certain model components are tied to specific government policies. We use our model to investigate the effect of specific interventions on supply chain performance.

**Table 1:** Examples of relationships between interventions impacting drug supply chain and the mathematical model parameters

Relationship between government interventions and model components	
**Government intervention**	**Model component**
Investment in prevention such as immunization programs	Population demand (*D*(*t*))
Building of new roads and highways, installation of railroads	Transportation time (*τ*)
Hiring of more personnel	Shipment periodicity (*n*)
Usage of higher capacity vehicles such as trains	Shipment size (*A* and *B*)

In some cases, one intervention can impact multiple model components

### Effect of installing a digital tracking system

The absence of essential drugs from health facilities may lead to detrimental health consequences on the population such as worsening of a health condition or even death. Hence, preventing drug stockouts is a major objective of drug supply chains.

The mathematical model can be solved analytically and a parametric relationship that prevents the depletion of drugs at facility level over time can then be derived:

6 $$  \frac{A_{WH}B_{WH}\sqrt{\pi}}{n_{WH}}=\overline{D},  $$

where $\overline {D}$ is equal to:

$$\overline{D}=\frac{1}{n_{WH}}\int\limits_{\tau_{WH}}^{\tau_{WH}+n_{WH}} D(t)dt. $$

If Eq. 6 is satisfied, drugs at the facility level would undergo a periodic variation over a constant level. However, direct implementation of new government interventions to satisfy condition (6) is not possible because the demand function is not known ahead of time; an approximate solution can be found if historical supply chain data is available. Furthermore, in some instances, a facility may be interested in procuring lower quantities of drugs to diminish its stocks in order to avoid drug expiration or to match an immediate budget drop. Hence, we propose to study the case of installing an automated system that keeps track of drug levels and patient demand. Such an initiative was recently taken by the Ethiopian Pharmaceuticals Supply Agency (EPSA), the primary provider of pharmaceutical services and essential medicines in Ethiopia, which began the implementation of the Integrated Pharmaceuticals Logistics System (IPLS) in 2009 in partnership with USAID, Supply Chain Management Systems (SCMS), and others [19].

**Fig. 3:**
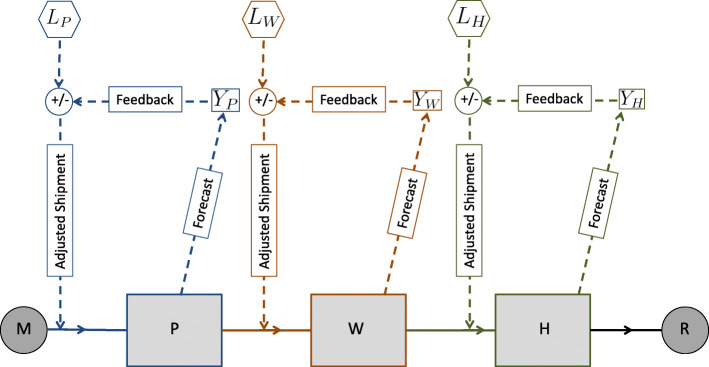
Simplified schematic of the forecasting technique. It is applied to the management of the supply chain of essential drugs in a low- and middle-income country setting

In our modeling, we implement feedback loops into the dynamical system, which utilize available information on drug quantities to forecast future stock levels and consequently offer an adaptive and continuous decision making process regarding required shipment sizes to keep inventory at desired levels, terminate stockouts and avoid drug expiration. We first introduce relevant ‘forecasting’ parameters: we define *L* as the desired stock level and *d* as the number of days before a shipment is scheduled when its size is determined. For example, *d*_*W*_=2 indicates that the size of a shipment leaving the warehouse is calculated two days before the scheduled shipment date, and *L*_*H*_=50 indicates that the quantity of drugs at health facilities should not be less than 50 packages at any time. A representative schematic of the forecasting methodology is given (Fig. 3). For each compartment, the stock level is forecasted into the future and is then sent back as a feedback to the supplier of that compartment; for example, the predicted stock level at health facilities is sent back to warehouses. The predicted stock level is then compared to the desired one, and the shipment size is adjusted. Details on the forecasting methodology follow.

We explain the forecasting process by considering an example of shipments from warehouses to health facilities in a given country ‘C’, with a selected choice of model parameters (Table 2).

**Table 2:** Relevant parameters for the modeling of the forecasting process in a given low- and middle-income country C

Country C							
*τ*_*WH*_	3 (days)	*n*_*WH*_	14 (days)	*L*_*H*_	50 (pkgs)	*d*_*W*_	2 (days)

**Fig. 4:**
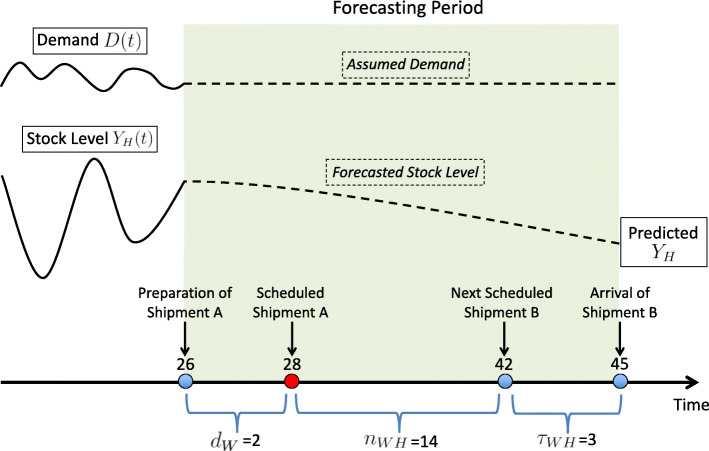
Demonstration of the forecasting period. The plots of functions *D*(*t*) and *Y*_*H*_(*t*) are meant for illustrative purposes

Country C would have shipment A scheduled on day 28 from warehouses to health facilities (Fig. 4). The size of this shipment would be determined *d*_*W*_=2 days before shipping (i.e. day 26). The next shipment B would be scheduled *n*_*WH*_=14 days later and would arrive on day 28+*n*_*WH*_+*τ*_*WH*_=45 (Fig. 4). Shipment A’s size should be sufficient to prevent *Y*_*H*_ from dropping below *L*_*H*_ in the period leading up to shipment B’s arrival. Hence, the forecasting period would span the period between days 26 and 45 (Fig. 4). We use our model to predict the stock level *Y*_*H*_ at the end of the forecasting period if no shipment is sent. When making such prediction, we assume that the demand rate *D*(*t*) would be constant throughout the forecasting period (Fig. 4). The predicted value of *Y*_*H*_ would then be compared to the desired value *L*_*H*_ to deduce the necessary shipment size. If *Y*_*H*_ becomes greater than *L*_*H*_, no shipment would be necessary, and if *Y*_*H*_ would be lower than *L*_*H*_, shipment A’s size would simply be their difference (*L*_*H*_−*Y*_*H*_). A similar methodology is applied regarding shipments between the other compartments. Such an intervention is specific to supply chains and as shown later would improve its performance substantially. Because the supply chain is connected to other health system elements, such improvement in drug delivery is translated into several other benefits such as decreasing mortality and morbidity.

## Results

The system of ordinary differential equations presented above was discretized and solved numerically using Matlab (version R2018b). We first examine how the stock levels at procurers, warehouses and health facilities would vary over time using an illustrative example of a low-income country. All parameter values are chosen for demonstration purposes and cover a range of values, yet they are not directly extracted from data. Later, we show how different government interventions that could be implemented by this illustrative country could affect drug levels; and we also study the implementation of a digital tracking system to study how drug stockouts and expiration could be anticipated and prevented.

### Example of a drug supply chain system in a low income country

**Fig. 5:**
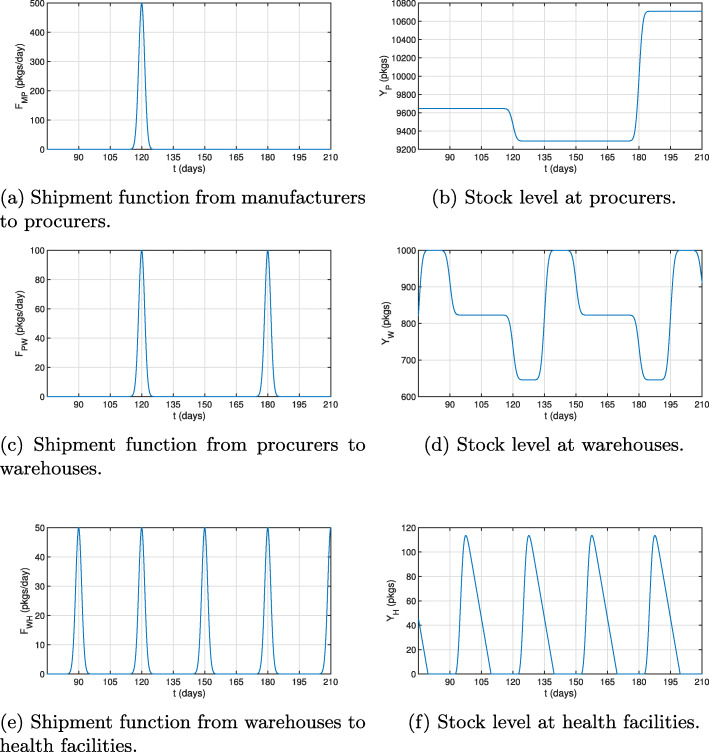
Shipment functions from, and stock levels at, the different compartments

We study the evolution of drug quantity at the different levels of the supply chain of a country for a chosen time period. All parameter values are given in Additional file 1,^1^. Stock level *Y*_*P*_ remains constant until a shipment leaves the procurers for the warehouses on day 120 as shown in Fig. 5c. This leads to an immediate decrease in the stock level *Y*_*P*_ (Fig. 5b). We observe that the stock level at the procurers increases on day 180, which is due to a shipment from the manufacturers that left on day 120 (Fig. 5a). The delay in the response of the stock levels is due to the transportation time *τ*_*MP*_ which is equal to 60 days.

Similar behaviors could be observed at warehouses. Instant drops in *Y*_*W*_ would coincide with shipments from warehouses to health facilities. However, there is a delay of fifteen days between shipments from procurers and changes in warehouse drug stock levels. For example, on day 120, a decrease in *Y*_*W*_ would occur (Fig. 5d) because of a shipment to health facilities on that day (Fig. 5e). The stock level would increase however on day 135 because of the arrival of a shipment from procurers that would also be sent out on day 120 (Fig. 5c). As for the stock levels at health facilities, it would change continuously because of the patient continuous demand *D*(*t*) (Fig. 5f). Every increase in *Y*_*H*_ would correspond to the arrival of a shipment from warehouses. As can be seen, with the current schedule of shipments, frequent stockouts would occur at health facilities. In the next section, we study the effect of different government interventions on drug levels and stockouts.

### Impact of selected drug supply chain interventions

We now use our mathematical model to examine the impact of interventions on the drug supply chain and the evolution of drug stocks at different levels in the considered country. Specifically, we study how the stock levels at health facilities would change over time from those displayed in Fig. 5f after a particular intervention is applied. The effect of the intervention on the incidence of stockouts is of particular interest. Because implementation of different interventions leads to different outcomes, economic evaluations would need to be performed to assess gains, in say drug quantity, per budget expenditure (i.e. per $ spent), for each intervention so to possibly implement the most cost-effective intervention in the end. For the purpose of this work, we select a few interventions and examine the way these interventions would affect drug stock levels. Details on the numerical implementation is provided in Additional file 1.

**Fig. 6:**
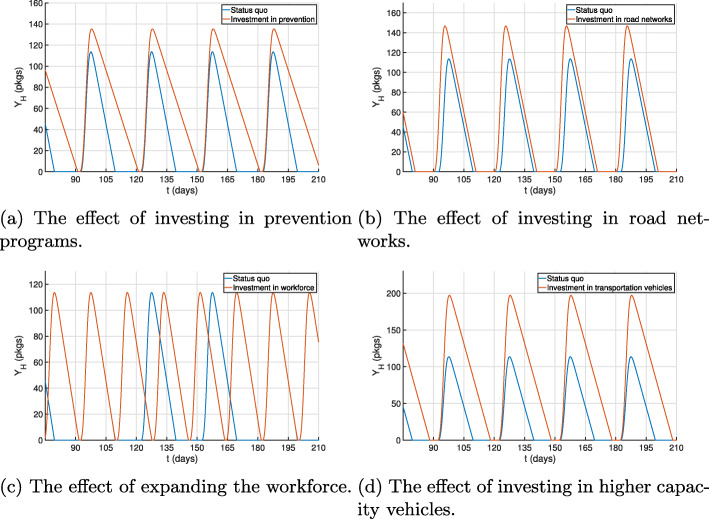
The impact of different supply chain interventions on health facility drug levels

First, we present the case of a government investing in vaccinations, where we assume it leads to a decrease in the number of individuals and patients needing drugs in the long term. We model this by reducing the demand function. As shown in Fig. 6a, the decrease in the uptake of medicines would lead to the accumulation of larger stocks of drugs at all times for the same supply chain system. This would lead to a decrease in the stockout period from 13 days to 1 day between shipments. Second, we consider an investment in improving the road network, which would be modeled by decreasing transportation times of drugs from warehouses to facilities. This change would also be accompanied by an increase in shipment sizes to model the arrival of shipments to facilities that were inaccessible prior to the intervention. The improvement in the drug level at health facilities would be minimal (Fig. 6b). Third, expanding the workforce would enable having more frequent shipments and would also have a positive impact on the drug stock levels (Fig. 6c); stockout periods would readily be terminated by the arrival of new shipments. Lastly, investing in purchasing transportation vehicles is examined. Although such intervention can result in a higher shipment frequency or size, we only consider here the latter: as one would predict, increasing shipment size would lead to higher drug quantities (Fig. 6d). We model this by increasing the shipment amplitudes.

### Prevention of drug stockouts through implementation of a digital tracking system

**Fig. 7:**
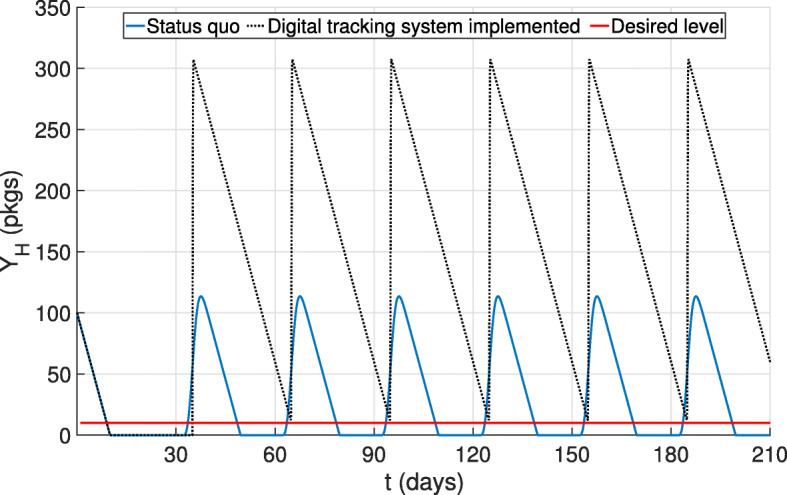
Adding the digital tracking system. The system keeps inventory at desired stock levels, terminates stockouts and avoids drug expiration

Similarly to stockouts, stock-ups of certain products may also be unfavorable due to the high costs associated with purchasing the drugs and to their expiration. A digital tracking system that comprises feedback loops and a forecasting technique as explained above is now implemented for the country under study. The goal here is to adapt the shipments to the demand function in such a way to keep drug stocks at desired levels, consequently avoiding stockouts and product expiration. If, for example, the desired drug level at health facilities was at least 10 packages, the drug quantity after applying the forecasting routine would always be above such desired level (Fig. 7). In addition, the drug quantity would be enough to prevent stockouts without additional costs of unneeded drugs that might expire.

**Fig. 8:**
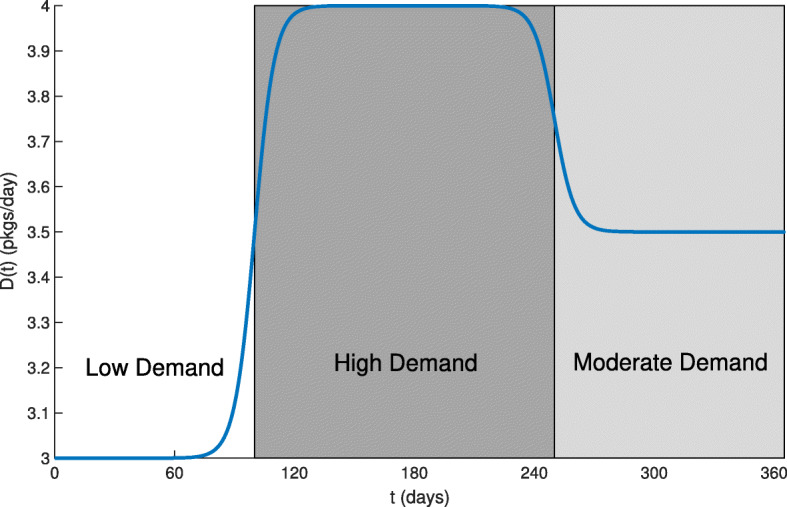
Transient demand function *D*(*t*) modeled over a full year

In order to illustrate the importance of the digital system, we study the case of a country experiencing a disease with a varying severity throughout the year. Figure 8 shows the drug demand function *D*(*t*) expected for the year ahead. The three distinct seasons are differentiated by background colors: the white background corresponds to the low season where demand *D*(*t*) for drugs is low; while the light and dark gray backgrounds correspond to moderate and high seasons, respectively, and where *D*(*t*) is larger.

**Fig. 9:**
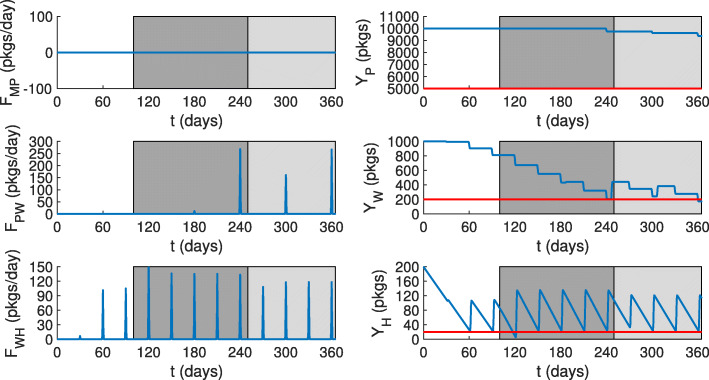
The effect of the digital tracking system on shipments from, and drug levels at, all model compartments

All relevant model parameter values for this case study are shown in Additional file 1. Our forecasting methodology could dynamically predict the required shipment sizes that would keep the drug stocks at the requested levels (Fig. 9). For example, as the stocks at health facilities decrease initially, sizes of shipments from warehouses would increase to push health facility drug quantities above *L*_*H*_. On day 100, when the high season starts (Fig. 8), the stock levels at health facilities would drop suddenly below *L*_*H*_ because of the insufficient shipment size. However, the implemented forecasting model could incorporate the new demand and could predict a higher shipment size for the next scheduled shipment which would readjust *Y*_*H*_ according to the desired *L*_*H*_. At the end of the high season and the start of the moderate season (day 250), shipments from warehouses to health facilities would drop in size. This in turn would cause stocks *Y*_*W*_ to drop at a slower rate. In regards to shipments from procurers to warehouses, no shipments would be sent for the first 180 days due to the sufficient stocks at warehouses compared to the desired level. A small shipment would be sent on day 180 and would be enough to prevent warehouse drug quantities to go below the desired one. Larger shipments to warehouses would follow. Finally, although shipments from manufacturers to procurers are scheduled every *n*_*MP*_=180 days, no shipments would occur. This is a result of the predicted stock levels being higher than the desired stock levels, meaning that no shipments would be required. As can be seen, shipments obtained from the forecasting routine would not be overestimated. In other words, they would result in quantities of drugs that are consumed and do not expire. This can be seen by the proximity of the stock levels to the desired stock levels (shown in red in Fig. 9).

## Discussion

Global health research has often focused on studying vertical programs driven by single interventions such as the introduction of a new vaccine or a new technology. However, national policymakers and international agencies (e.g. Gavi, the Vaccine Alliance; the Global Fund To Fight AIDS, TB, and Malaria) are paying increasing attention to strengthening health systems and to improving their efficiency [18]. In addition to solely investigating disease-focused interventions, for example, analysts need to examine system-wide delivery platforms such as health facilities, and operational elements such as supply chains. In this paper, we adopted such a new paradigm and implemented it computationally to test how supply chains could strengthen existing health systems.

We developed a mathematical model composed of simple differential equations that incorporate some of the constraints limiting the improvement of population health. Such mathematical tools can model the stock levels of essential drugs at procurers, warehouses and facilities and afford the chance of investigating multiple HSS interventions in order to propose cost-effective strategies and interventions. Several interventions were considered here, namely investing in prevention programs (e.g. immunization programs, behavioral risk factor control) to lower the population need for treatment (e.g. drugs), improving the quality and design of roads, recruiting more personnel to conduct delivery and distribution of essential drugs, and purchasing additional transportation trucks to move higher loads of drugs or building train rails. In addition, we explored how implementing a digital tracking system of drugs with an internal forecasting routine would be effective in controlling drug flows and prevent drug stockouts and overall supply chain bottlenecks. This enables the quantification of stock levels at health facilities at any given time, which would inform high-level decisions about procurement quantities and shipment needs in real-time.

There is a growing movement towards modeling health systems to identify bottlenecks and solutions with a more holistic understanding of how interventions in one area can drive impacts on others. This understanding can support horizontal improvements for health system efficiency and outputs. As a component of a larger, complex system, the pharmaceutical supply chain can respond to and influence features such as disease burden (e.g., investment in prevention interventions, plateauing prevalence as a result of adequate treatment), patient demand (e.g., changes patients’ information or education about pharmaceutical treatments), developments in medical education (e.g., provider knowledge about certain medicines), allocation of human resources, use of medical technology, and infrastructure investment and planning, for example. As a result, this model of the pharmaceutical supply chain can act as a component of a larger model, in which other components may represent the allocation of providers based on patient demand and disease burden, expected or changing trends in the production of medical professionals, technological interventions, or the expansion of a national social protection scheme.

Use and integration of this model into analyses of health systems and proposed interventions can allow for measurement of unintended consequences of interventions across the health system. Not only does it allow for greater understanding of the system through description and simulated testing of interventions, but can also support goal setting. Mathematical modeling lends itself to optimization techniques. Given a set of target parameters, optimization analyses would be able to identify which other features (that presumably the government, organization, or other implementing agency has control over) to intervene on to successfully reach targets. Alongside these gains, there are inevitably limitations in the use of models to represent real-world impacts. Although systems dynamics models such as the one developed in this paper may articulate assumptions about how a particular system works or illuminate the high-level effects of certain policies, they are not intended to illustrate the behavior of individuals within a system. In this regard, systems dynamics models tend to be viewed as strategic models [20, 21]. Other modeling methods, such as discrete event simulations, tend to be more useful for operational purposes, where there is a need for better numerical estimates and predictions [20, 21]. In addition, it is necessary that researchers and other users understand the quality of parameter inputs used and to be aware of all model assumptions oand their effects on results. Understanding how sensitive the model is to any of these assumptions would be critical to assessing an intervention.

Through our mathematical approach, we could investigate the effect of multiple interventions on the supply chain of essential medicines. However, more experiments that cover a wider range of interventions may be conducted in order to validate the model further and allow more confidence in its results. Further, our interpretation of the findings could be strengthened by validating the model against data. Future work will test our model using multiple global health datasets. Specifically, we will apply our mathematical framework to the Ethiopian health system. We will consider interventions such as increasing the efficiency of pharmaceutical procurement and delivery, and training and deployment of workforce, in order to investigate their effects on improving health and its distribution across the population of the country. Such a task requires to build and improve from the current model; characterization of the health system infrastructure (e.g. services, facilities), the distribution and allocation of human resources (e.g. health vs. non-health workforce), and the contextual networks impacting on health service delivery (e.g. roads, population locations) would be required in order to extract the relevant model parameters. For example, datasets on GPS locations of different facilities, consumption patterns at facilities, population distribution, outpatient and inpatient visits are all important factors that would need to be accounted for.

## Conclusions

The ability to model the flow of essential medicines in low- and middle-income country settings and to suggest reform policies pertaining to supply chain and human resources is an essential step towards HSS. New mathematical models connecting the multiple elements of health systems along with novel approaches in the conduct of economic evaluations are needed to help policymakers choose what may be good value for money investments for HSS interventions, including improvement of supply chain management.

## Supplementary information

**Additional file 1** This file contains a summary of all model parameters and their definitions. In addition, the parameters’ numerical values used to conduct the presented simulations are all given.

## Data Availability

Not applicable.

## Abbreviations

LMIC: Low- and middle-income country
JIT: Just in time
HSS: Health system strengthening
NGO: Non-governmental organization
EPSA: Ethiopian pharmaceuticals supply agency
IPLS: Integrated pharmaceuticals logistics system
SCMS: Supply chain management system

## Footnotes

1 In this computation, parameters *d* and *L* are not involved.
