# Supplementary material for: Toward health system strengthening in low- and middle-income countries: insights from mathematical modeling of drug supply chains
Source: BMC Health Serv Res. 2020 Aug 24;20:776. doi: 10.1186/s12913-020-05549-z (PMC7445921; doi:10.1186/s12913-020-05549-z)
Supplement: Supplementary file 1 — Additional file 1 This file contains a summary of all model parameters and their definitions. In addition, the parameters’ numerical values used to conduct the presented simulations are all given. [file 12913_2020_5549_MOESM1_ESM.pdf]

Additional file 1 — Definitions and values of model parameters.

This file contains a summary of all model parameters and their definitions. In addition, the parameters' numerical values used to conduct the presented simulations are all given.

Parameters for the example on drug supply chains

The model parameters used in the section entitled *"Example of a drug supply chain system in a low income country"* are listed in table 1. An average demand function $D(t) = 10$ (packages per day) is considered. Initially, the stock levels at the procurers, warehouses and health facilities were set to 10,000 (packages), 1000 (packages), and 100 (packages), respectively.

Table 1: Model parameter values assigned for modeling the drug supply chain in a low- and middle-income country.

Input parameter values for the drug supply chains							
τ_{MP}	60 (days)	n_{MP}	120 (days)	A_{MP}	500 (pkgs/day)	B_{MP}	2 (days)
τ_{PW}	15 (days)	n_{PW}	60 (days)	A_{PW}	100 (pkgs/day)	B_{PW}	2 (days)
τ_{WH}	5 (days)	n_{WH}	30 (days)	A_{WH}	50 (pkgs/day)	B_{WH}	2 (days)

Parameters for the different interventions studied

In the section entitled *"Impact of selected drug supply chain interventions"*, the intervention of investing in prevention programs is modeled by a 40% decrease in the demand function for drugs.

The intervention of investing in road networks is modeled by a 30% decrease in transportation times from warehouses to health facilities. In order to include the possibility of having access to new facilities through newly build roads, the shipment amplitude is increased by 20%.

The intervention of expanding the workforce is modeled by repeating shipments from warehouses to facilities every 18 days instead of 30.

The intervention of investing in transportation vehicles is modeled by increasing warehouse shipments by 50%.

Parameters for the study of implementing the digital system

The model parameters used in the section entitled *"Prevention of drug stockouts through implementation of a digital tracking system "* are listed in table 2. Initially, the stock levels at the procurers, warehouses and health facilities were set to 10,000 (packages), 1000 (packages), and 200 (packages), respectively.

Table 2: Model parameter values assigned for modeling the drug supply chain in a low- and middle- income country.

Assumed parameter values for the drug supply chain					
τ_{MP}	90 (days)	τ_{PW}	7 (days)	τ_{WH}	2 (days)
n_{MP}	180 (days)	n_{PW}	60 (days)	n_{WH}	30 (days)
B_{MP}	0.25 (days)	B_{PW}	0.25 (days)	B_{WH}	0.25 (days)
d_{MP}	2 (days)	d_{PW}	2 (days)	d_{WH}	2 (days)
L_P	5000 (pkgs)	L_W	200 (pkgs)	L_H	20 (pkgs)
